# New Strategies in the Design of Paramagnetic CAs

**DOI:** 10.1155/2020/4327479

**Published:** 2020-09-27

**Authors:** Alessio Smeraldo, Paolo A. Netti, Enza Torino

**Affiliations:** ^1^Department of Chemical, Materials Engineering & Industrial Production, University of Naples Federico II, Piazzale Tecchio 80, Naples 80125, Italy; ^2^Center for Advanced Biomaterials for Health Care, CABHC, Istituto Italiano di Tecnologia IIT@CRIB, Largo Barsanti e Matteucci 53, Naples 80125, Italy; ^3^Interdisciplinary Research Center on Biomaterials, CRIB, Piazzale Tecchio 80, Naples 80125, Italy

## Abstract

Nowadays, magnetic resonance imaging (MRI) is the first diagnostic imaging modality for numerous indications able to provide anatomical information with high spatial resolution through the use of magnetic fields and gradients. Indeed, thanks to the characteristic relaxation time of each tissue, it is possible to distinguish between healthy and pathological ones. However, the need to have brighter images to increase differences and catch important diagnostic details has led to the use of contrast agents (CAs). Among them, Gadolinium-based CAs (Gd-CAs) are routinely used in clinical MRI practice. During these last years, FDA highlighted many risks related to the use of Gd-CAs such as nephrotoxicity, heavy allergic effects, and, recently, about the deposition within the brain. These alerts opened a debate about the opportunity to formulate Gd-CAs in a different way but also to the use of alternative and safer compounds to be administered, such as manganese- (Mn-) based agents. In this review, the physical principle behind the role of relaxivity and the *T*_1_ boosting will be described in terms of characteristic correlation times and inner and outer spheres. Then, the recent advances in the entrapment of Gd-CAs within nanostructures will be analyzed in terms of relaxivity boosting obtained without the chemical modification of CAs as approved in the chemical practice. Finally, a critical evaluation of the use of manganese-based CAs will be illustrated as an alternative ion to Gd due to its excellent properties and endogenous elimination pathway.

## 1. Introduction

Magnetic resonance imaging (MRI) is a diagnostic technique used to obtain anatomical images from the human body. Several advantages as noninvasiveness, no ionizing radiation, submillimetre spatial resolution, and precise 3D positioning ability are strengths of this modality. MRI is based on the intrinsic properties of hydrogen protons, which act like charged particles whose rotation generates a magnetic moment [1–3]. MRI principles, therefore, refer to ^1^H nuclei of the water molecules already present in the tissues. In normal conditions, magnetic dipoles are oriented randomly but, when exposed to a high external magnetic field *B*_0_ (clinically used field intensity is about 1.5–3 Tesla), they align in the same direction of the applied field. Therefore, dipoles orientation leads to a resultant vector, called Longitudinal Magnetization (LM), in the same direction of *B*_0_  (usually correspond to the *z*-axis). Protons are not perfectly oriented in a parallel direction, but they diverge from *B*_0_ of a certain angle that turns around the *z*-axis. This motion leads to a precession motion that is a clockwise rotation around the *z*-axis. Transverse Magnetization (TM) is zero value because each proton has a different phase. When a radiofrequency impulse is applied, if electromagnetic waves frequency is the same as precession frequency (called Larmor frequency), the result is the synchronization of protons' spin, which leads to two effects: LM becomes zero while TM increases. After the removal of the RF pulse, during initial condition return, a signal is produced, and it depends on two parameters: Transverse Relaxation Time (*T*_2_) and Longitudinal Relaxation Time (*T*_1_). The first is employed time in TM decay while the second one is employed time on LM rebuilding. Each tissue has its own relaxation times to return at the starting condition, and these times depend on tissue composition and status.

Thanks to differences in proton density, *T*_1_ or *T*_2_ relaxation time, and rates of water diffusion, MRI permits to discriminate between normal and pathological tissues. However, to enhance further the signal intensity and improve the contrast between distinct tissues, substances called contrast agents (CAs) are intravenously injected before MRI scans.

## 2. MRI Contrast Agents

The extensive use of contrast agents is based on their property to induce additional contrast to MRI image making visible anatomical details otherwise not appreciable. At molecular level, this enhancement is explained by the presence of unpaired electrons nearby water molecule that, having a magnetic moment 658 times higher than hydrogen proton, shorten the return to equilibrium position [4, 5]. To indicate the potency of a CA to reduce *T*_1_ and *T*_2_ relaxation times, the concept of relaxivity is introduced [5]. The relaxivity (*r*_1_ or *r*_2_) is defined as the change in the relaxation rate (ΔT) per unit of CA concentration ([*M*]) after its introduction in the body [5]:

(1) $$\begin{array}{c} r_{i}=\left(\frac{\Delta T}{\left[M\right]}\right)=\frac{\left(1/T_{i}\right)-\left(1/T_{i0}\right)}{\left[M\right]},\;i=1,2. \end{array}$$

**Figure 1:**
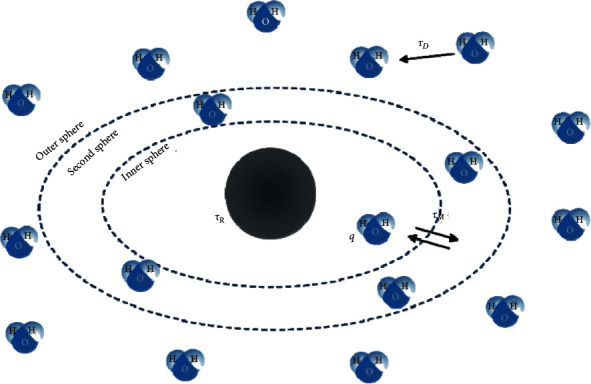
Schematic representation of the three spheres around the metal complex.

Water molecules into proximity of metal ions are involved in specific chemical interactions, which play an important role in the transmission of the relaxation effect to the bulk water [6]. Therefore, water molecules can be classified into three categories called “spheres” (see Figure 1) [5]:

  Inner Sphere (IS) water, where the water is directly coordinated to the metal ion
  Second Sphere (2ndS) water, in which water molecules have a finite residency time that is longer than the translational diffusion time of pure water
  Outer Sphere (OS) water, where the interaction is only due to translational diffusion

The total relaxivity is the sum of the relaxivity given by each sphere:

(2) $$\begin{array}{c} r_{i}=r_{i}^{\text{IS}}+r_{i}^{2\text{ndS}}+r_{i}^{\text{OS}}. \end{array}$$

Magnetic dipole fluctuations influence the relaxation process deeply. In particular, these fluctuations are described through three different correlation times, which will be described in the folllowing.

Obviously, many studies have been focused on the modeling of each sphere in order to foresee the relaxation rate and know how much they contribute to the total relaxivity. While the Solomon–Bloembergen–Morgan theory well describes the inner sphere mechanism, the modeling of the other two spheres is more complicated [6]. The Hwang–Freed's hard sphere is one of the models used for the outer sphere mechanism description, where relaxation is determined primarily by the diffusion coefficient of water and the distance of the closest approach [7]. Instead, the contribution of the second sphere can be calculated from expressions similar to the first sphere relaxation but, very often, this contribution is neglected or included in the outer sphere term. In the end, the inner sphere relaxivity can be considerably boosted thanks to a deeper knowledge about it and, consequently, it becomes more important in the development of new contrast agents.

### 2.1. Solomon–Bloembergen–Morgan Theory

The inner sphere term gives the major contribution to the overall relaxivity (more than 60%) and, for this reason, it has been deeply studied. Moreover, differently from the outer sphere, there are many parameters that can be handled and used in order to increase the total relaxivity. The inner sphere contribution arises from the chemical exchange of the coordinated water protons with the bulk. The longitudinal relaxation rate is given by the following equation:

(3) $$\begin{array}{c} {\left(\frac{1}{T_{1}}\right)}^{\text{IS}}=\left(\frac{P_{m}}{T_{1m}+\tau_{m}}\right)=\left(\frac{q}{\left[H_{2}O\right]\left(T_{1m}+\tau_{m}\right)}\right), \end{array}$$

where *q* is the hydration number,  *P*_*m*_ is the mole fraction of bounded water molecules, [H_2_O] is the water mM concentration, *τ*_*m*_ is the lifetime into the inner sphere of a water molecule, and  1/*T*_1*m*_ is the longitudinal relaxation rate [5].

The Solomon–Bloembergen (SB) theory well describes the inner sphere contribution, although several severe approximations have been made [6, 8]. The two mechanisms involved in the relaxation are dipole-dipole (DD) and scalar (SC) (or contact) interaction [9]:

(4) $$\begin{array}{c} \frac{1}{T_{1m}}=\left(\frac{1}{T_{1}^{\text{DD}}}\right)+\left(\frac{1}{T_{1}^{\text{SC}}}\right),\\ \frac{1}{T_{1}^{\text{DD}}}=\left(\frac{2}{15}\right)\frac{g^{2}\gamma_{I}^{4}\mu_{B}^{2}\text{S}\left(\text{S}+1\right)}{r_{\text{GdH}}^{6}}{\left(\frac{\mu_{0}}{4\pi }\right)}^{2}\left[\frac{7\tau_{c2}}{\left(1+\omega_{S}^{2}\tau_{c2}^{2}\right)}+\frac{3\tau_{c1}}{1+\omega_{I}^{2}\tau_{c1}^{2}}\right],\\ \frac{1}{T_{1}^{\text{SC}}}=\frac{2S\left(S+1\right)}{3}{\left(\frac{A}{h}\right)}^{2}\left(\frac{\tau_{e2}}{1+\omega_{S}^{2}\tau_{e2}^{2}}\right), \end{array}$$

where *g* is the electron *g* factor (isotropic assumption), *μ*_*B*_ is the Bohr magneton, *r*_GdH_ is the electron spin-proton distance, *ω*_*S*_ and *ω*_*I*_ are the electron and nuclear Larmor frequency, *γ*_*I*_ is the nuclear gyromagnetic ratio (*γ*_*s*_ = 658.2  *γ*_*I*_), and A/h is the hyperfine constant between the electron spin and the water proton.

The correlation times can be split into the following:

(5) $$\begin{array}{c} \frac{1}{\tau_{\text{ci}}}=\left(\frac{1}{\tau_{\text{R}}}\right)+\left(\frac{1}{T_{\text{ie}}}\right)+\left(\frac{1}{\tau_{m}}\right)\text{,}\;\;i=1,2,\\ \frac{1}{\tau_{\text{ei}}}=\left(\frac{1}{T_{\text{ie}}}\right)+\left(\frac{1}{\tau_{m}}\right)\text{,}\;\;i=1,2. \end{array}$$

Here, *τ*_*R*_ is the rotational correlation time of the metal ion-water proton vector, *T*_1*e*_ and *T*_2*e*_ are the longitudinal and transverse electron spin relaxation time of the metal ion, and *τ*_*m*_ is the lifetime of a water molecule in the inner sphere (it is the reciprocal of the water exchange rate *k*_*m*_). It is evident that correlation time is dominated by the shortest correlation time among these three, and it strongly depends on the magnetic field strength used to obtain information [5]. Usually, for clinical imaging, a field strength of 1.5 T is employed and the dominant correlation time is the rotational ones [9]. An approach to obtain an enhancement at this field is, indeed, slowing down rotation by increasing the molecular weight of the complex. Instead, at low fields (<0.1 T), electronic relaxation is the fastest and so it is the dominant correlation time. Finally, the water exchange must be neither too slow, since it is important to transmit the relaxation effect, nor too fast because it means that water is not coordinated enough with the metal ion.

It is also useful to consider the distance between the water proton and the metal ion centre to understand if the SC term has to be considered. In the case of Gd (III), the *r*_GdH_ value is about 3.1 Å and consequently, the SC contribution is very weak and so negligible, while this interaction might be important for Mn (II).

As mentioned before, the SB equations are based on some assumptions. For example, they are valid within the Redfield limit. The Bloembergen–Morgan theory, valid only at high field (Zeeman limit), defines electronic relaxation rates for metal complex with *S* ≥ 1 interpreted in terms of zero-field splitting (ZFS) interaction resolving the dependence of the electronic relaxation on ZFS time fluctuations [10]:

(6) $$\begin{array}{c} {\left(\frac{1}{T_{1e}}\right)}^{\text{ZFS}}=2C\left(\frac{1}{1+\omega_{S}^{2}\tau_{V}^{2}}+\frac{4}{1+4\omega_{S}^{2}\tau_{V}^{2}}\right),\\ {\left(\frac{1}{T_{2e}}\right)}^{\text{ZFS}}=C\left(\frac{5}{1+\omega_{S}^{2}\tau_{V}^{2}}+\frac{2}{1+4\omega_{S}^{2}\tau_{V}^{2}}+3\right),\\ C=\left(\frac{1}{50}\right)\Delta^{2}\tau_{V}\left\{4S\left(S+1\right)-3\right\}, \end{array}$$

where Δ^2^ is the mean squared fluctuation of the ZFS and *τ*_*V*_ is the correlation time for the modulation of the ZFS.

Therefore, the SBM theory is the combination of the SB and Morgan's electronic relaxation equations, which well represent the connection between the microscopic properties and the observed relaxation rate and it is the more appropriate contribution to the study of old and new CAs.

### 2.2. Outer and Second Spheres

As mentioned before, the second sphere contribution is mostly included in the outer sphere term, but they are calculated differently. The latter is mainly described by translational diffusion due to the Brownian motion of free water molecules that could faintly interact with the electronic spins of the metal ion through dipolar intermolecular interactions. The water and metal complex diffusion coefficients can be estimated using a model, developed by Hwang and Freed, consisting of rigid spheres, having a molecular radius *α*_*i*_, in a medium with viscosity *η*:

(7) $$\begin{array}{c} D_{i}=\left(\frac{\text{KT}}{N_{A}6\pi \alpha_{i}\eta }\right),\;i=\text{I},\text{S}. \end{array}$$

where *N*_*A*_ is the Avogadro's number. The relative translational diffusion time can be expressed as follows:

(8) $$\begin{array}{c} \tau_{D}=\left(\frac{d^{2}}{3\left(D_{I}+D_{S}\right)}\right)=\left(\frac{d^{2}}{3D}\right). \end{array}$$

The term *d* represents the distance of the closest approach of spins S and I. In the end, the outer sphere relaxivity is given by the following:

(9) $$\begin{array}{c} {\left(\frac{1}{T_{1}}\right)}^{\text{OS}}=\left(\frac{32\pi }{405}\right)\frac{\hbar^{2}\gamma_{I}^{2}\gamma_{S}^{2}\text{S}\left(\text{S}+1\right)N_{A}\left[M\right]}{\text{dD}}{\left(\frac{\mu_{0}}{4\pi }\right)}^{2}\left[3j\left(\omega_{I},T_{2e},\tau_{D}\right)+7j\left(\omega_{S},T_{1e},\tau_{D}\right)\right]. \end{array}$$

Instead, the second sphere contribution is the result of several and different types of binding sites interactions. Consequently, the contribution to the longitudinal relaxation is as follows:

(10) $$\begin{array}{c} r_{1}^{2\text{nd}}=\left(\frac{10^{-3}}{55.55}\right)\sum_{j=1}^{M}\frac{q_{j}^{2\text{nd}}}{T_{1j}^{2\text{nd}}+\tau_{\text{mj}}^{2\text{nd}}}, \end{array}$$

where *q*_*j*_^2nd^ is the number of water molecules in a specific site *j* with a lifetime *τ*_mj_^2nd^ and *T*_1*j*_^2nd^ is its relaxation time that can be estimated using the inner sphere equations.

## 3. Gadolinium-Based CAs

Since electrons have a higher magnetic moment than protons, lanthanides represent the best way to improve relaxivity thanks to their unpaired electrons. In particular, Gadolinium (Gd^3+^) contains seven unpaired electrons that lead it to be the most widespread metal ion used in complex contrast agents. Even though other lanthanides with a higher magnetic moment exist, Gadolinium has an optimal electron spin relaxation and it is able to form very stable molecules. In fact, free Gd^3+^ ions are toxic and the chelation can reduce side effects. Among ligands used for Gadolinium complexation, DOTA (dodecane tetraacetic acid) (CA trade name: Dotarem®) and DTPA (diethylenetriaminepentaacetic acid) (CA trade name: Magnevist®) are two common macrocyclic ligands owing a kinetic inertness that reduces the risk of complex dissociation *in vivo*.

However, the enhancement of the relaxivity that is possible to get with Gd-CAs is also reduced by the chelation of the metal ion. In fact, octadentate chelates are present in all clinically approved Gd-based CAs and, since the coordination number of the Gd^3+^ is nine, this leads to one available coordination site for water, reducing the possible relaxivity drastically.

### 3.1. Gadolinium Safety

Gd-CAs are widely used in clinical practice but, in the last years, a focus on their associated risks has pointed out a triggering role in the development of particular nephrogenic system fibrosis (NSF) [11, 12]. In fact, it was observed that patients with renal disease, after some MR angiographies, had to undergo dialysis for renal failure. Afterward, indirect evidence showed that Gadolinium deposition may also occur in patients with normal renal functions with particular attention to the brain [13, 14]. The mechanism used by Gadolinium, associated or not with the ligand, to cross the blood-brain barrier (BBB) remains unclear, but it has been highlighted that deposition may also occur in the presence of an intact BBB. Therefore, the cellular response to Gd exists since the observed deposition is not uniform in neuronal tissue [15]. In particular, the regions involved are the dentate nucleus and globus pallidus that show a brighter signal in unenhanced *T*_1_-weighted MR images [13, 16]. A correlation between the number of Gadolinium-based CAs intravenous administration and the deposition in the brain has been demonstrated while no dependence on age, weight, sex, and renal function status has been found [13, 15].

Gd-CAs toxicity is mainly related to free *Gd*^3+^ions that are slowly excreted from the body and compete biologically with *Ca*^2+^ ions [17–19]. The dissociation of the chelating agent from the metal ion is termed transmetallation. This phenomenon stems from the interaction of the *Gd*^3+^ or the ligand with endogenous anions and cations, which may destabilize the Gadolinium complex and lead to free components [17, 18]. It strongly depends on the kinetic and thermodynamic stabilities of the Gd-CAs. In fact, there is an evident difference between macrocyclic and linear chelating agents. Moser et al. demonstrated that a significant signal intensity boost in dentate nucleus and globus pallidus was present in patients receiving linear agent group while poor changes were detected in the group receiving gadobutrol indicating greater stability of this latter [16].

### 3.2. Gd-Based Nanostructures to Boost T1 Signal

As already stated in the previous paragraph, despite the widespread use of Gadolinium-based CAs in clinical practice, they still suffer from many drawbacks. One of the main limitations is the lack of tissue specificity that leads to having low sensitivity in the investigated region and relaxivity far below their theoretical limit. Moreover, the technological progress, represented by MRI scans at high magnetic fields able to give back high-resolution images, is not supported by efficient CAs. In this sense, two crucial issues are represented by the decrease of Gadolinium-based CAs longitudinal relaxivity when the magnetic field intensity grows and to the toxicity of these CAs, due to possible dechelation *in vivo* [20, 21].

Recent examples have proved that it is possible to entrap Gd-based CAs within nanostructures to improve relaxivity without metal complex chemical modification [22–31]. In some cases, as a result of this “peculiar” encapsulation of the CAs, it has also been reported that the characteristic correlation times, as described by the SBM theory, can be strongly modified. In particular, it is possible to handle nanostructure properties in order to obtain the desired boost in relaxivity.

Initially, Port et al. have proved the impact of rigidification on the longitudinal relaxivity of Gd–PCTA12 due to the replacement of one ethylene bridge with a cyclohexylene bridge to form Gd-cyclo-PCTA12 [32]. The rigidity of the metal complex induces a shortening of the residence lifetime *τ*_*m*_ (82 vs. 34 ns at 310 K) of the water molecule in the inner sphere resulting in a higher relaxivity. Later, Sethi et al. have examined the performance of Gd-DTPA when it is geometrically confined within mesoporous silica microparticles without involving any chemical modification of the metal complex [22]. They observed an enhancement of the relaxometric properties explained by the influence of SBM parameters, *τ*_*D*_ and *τ*_*R*_ , due to the geometrical confinement within nanopores. Indeed, *τ*_*D*_ goes through a change since the mobility of water molecules is very different to bulk phase in confined conditions [22–24, 33]. In fact, additional interaction forces, arising at water-solid interfaces, reduce local diffusion and lead to defining a self-diffusion coefficient *D* of water, which depends on geometrical and physicochemical parameters [33]. The second correlation time, *τ*_*R*_, go through a change because Gadolinium complexes cannot tumble freely because they are absorbed on the walls of the pores [22–24].

**Figure 2:**
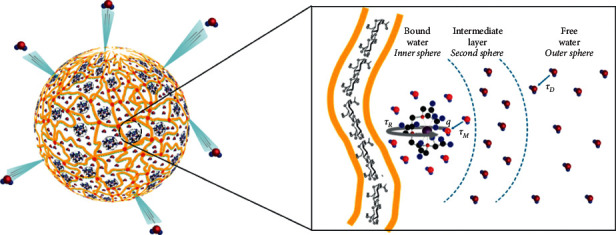
Graphical representation of the influence of crosslinked polymer matrix on water molecules dynamics.

Among the available nanostructures to carry CAs, polymeric-based nanocomposites have widely captured the attention of researchers thanks to their properties like biocompatibility, biodegradability, and no toxicity [34, 35]. Further advances in the *T*_1_ boosting have been reported using biocompatible hydrogels, taking advantage of their hydrophilicity. Indeed, hydrogel matrices made up of hydrophilic polymers are able to accumulate a large amount of water inside the structure, increasing interactions between water molecules and the metal chelate and, consequently, promoting a relaxivity boosting of the T1 CAs [25, 26, 28–30]. The effect has been very well described by Russo et al. in the Hydrodenticity concept [31]. They synthesized crosslinked Hyaluronic Acid NPs (cHANPs) loaded with Gd-DTPA through a microfluidic flow-focusing process, demonstrating the possibility to improve relaxivity through the tunability of crosslink density, mesh size, hydrophilicity, and loading capability and by changing the process parameters [30]. Indeed, the proper control of the structural properties of polymer-based nanohydrogels affects the water molecules' dynamics resulting in a specific condition in a relaxivity boost [36, 37]. This effect is called *Hydrodenticity* [31]. In particular, when a complex equilibrium between elastodymanic forces of the polymer chains, water osmotic pressure, and hydration degree of Gd-CAs is reached, SBM correlation times go through a change [28]. The improved relaxation rate is the result of an increased residence lifetime of water molecules within the crosslinked polymer matrix, a restricted molecular tumbling, and a resulting faster exchange rate with metal ions (see Figure 2).

Moreover, adding a second polymer in the hydrogel matrix, it is possible to introduce a further tuning of the parameters to control the *Hydrodenticity* and release properties of the architectures. Vecchione et al. produced Chitosan-core and Hyaluronic Acid-shell nanoparticles through a complex coacervation method for multimodal imaging and theranostic applications [27]. Integration of Gadolinium chelates into NPs and particularly in polyionic nanocomplexes, composed of polyanions and opposite polycations, allows controlling water exchange and reducing Gd-DTPA concentration compared to other CAs [38]. In fact, several examples of NPs obtained through ionotropic gelation between chitosan and hyaluronic acid exist in the literature, showing how much the relaxivity is improved [25–27, 39]. Using the same chemical principle is possible to combine also chitosan and alginate to create a drug or CAs-delivering hydrogel [40–45]. Instead, Chen et al. developed PLA-PEG nanoparticles for liver MRI where Gd-DTPA is attached to the surface, taking advantage of the rigidification but inducing a chemical modification of the approved compounds, therefore, potentially compromising its stability *in vivo* [46].

In conclusion, the use of biopolymers provides us with an extensive library of polymers to create functional nanocarriers for enhanced imaging properties. Furthermore, these nanosystems offer the opportunity to perform a precision imaging by functionalizing their surface with selected ligands (peptide, antibody, etc.) to guide the CAs to a specific target or decorated with other polymers (e.g., PEG) to extend their permanence in the bloodstream [47–49].

## 4. Manganese-Based CAs

Manganese (Mn) represents, potentially, an additional opportunity to CAs for MRI, able to overcome some of the previously described drawbacks. The main reasons behind this are two: (1) manganese is an element already present in the human body, involved in many cellular processes and so free ions are rapidly taken up by cells such as hepatocytes, cardiomyocytes, and pancreatic tissue [50]. Therefore, the presence of an endogenous elimination pathway might be a great advantage but, however, determined Mn concentration levels must be respected; (2) Mn shows all the Gd physical properties: high spin quantum number (five unpaired electrons), long longitudinal electronic relaxation times, and faster water exchange kinetics. Moreover, differently than Gd, Mn has a low *T*_2_ at high field strength MRI that make it useful also in *T*_2_-weighted imaging [50–52].

However, also in this case, chelators were used to create more stable probes and to protect Mn to the rapid cellular uptake. MnDPDP (Teslascan®, DPDP = dipyridoxyl diphosphate) was the only FDA approved manganese-based CAs as a liver imaging agent [53]. This CA had a safety factor that was 5 times higher than Gd-DTPA. Nevertheless, due to poor clinical performance and overtoxicity, as a consequence of Mn-DPDP dephosphorylation and simultaneous transmetallation with zinc in the blood, it was withdrawn from the USA market in 2003 and successively from the EU market in 2012. Indeed, Mn-DPDP undergoes partial dechelation and consequent cellular uptake that lead to the need for a more stable and inert chelator. For this reason, Gale et al. recently developed a new chelator called PyC3A that leaves one coordination site for water, providing high relaxivity and resistance to Mn dissociation [54]. *In vivo* preliminary analysis on animals has shown good blood and kidney clearance and only intact Mn-PyC3A complexes have been detected in urine [54, 55].

The manganic dioxide and oxide nanoparticles have also attracted great interest in their high *T*_1_ relaxivity combined with the possibility to have a therapeutic effect thanks to the release of therapeutic drugs or as a result of external stimuli [56]. Fu et al. produced HA-Mn*O*_2_ NPs able to bind to CD44 receptors overexpressed in glioma cells and, at the same time, thanks to the response of Mn*O*_2_ to the tumor acid environment, produce *O*_2_ reducing tumor hypoxia [57]. Li et al. prepared PEG-MnO NPs as *T*_1_ CAs with a *r*_1_ value (12.942 s^−1^mM^−1^) 3 times higher than commercial Gd-DTPA (4.2 *s*^−1^m*M*^−1^) and, furthermore, a ratio *r*_2_/*r*_1_ = 4.66 at 3.0 T, which means that it is really efficient as a negative CA too [58]. This behaviour might be due to the high concentration of Mn onto NPs nanoparticles and the increased hydrophilicity promoted by PEG carboxyl groups. These new nanostructures represent a promising opportunity for the use of MnO_2_.

Furthermore, other formulations based on Mn have been proposed following the current strategy to use higher magnetic fields to achieve a better signal-to-noise ratio and reduce acquisition times [59]. Indeed, *T*_1_ relaxivity decreases the above clinical used 1.5 T field strength while *T*_2_ relaxivity raises and Mn owns a better transverse relaxivity than Gd [21, 60]. For this reason, combinations of Mn with specific magnetic molecules to create *T*_1_/*T*_2_-contrast agents able to satisfy this requirement have been proposed [61–65]. Sana et al. developed a magnetic manganese-ferritin nanocomposite (MnAfFtn-AA) with high relaxivity values *r*_1_ and *r*_2_ [66]. In particular, the second one is higher due to the conjugation with large protein that leads to the enhancement of the scalar mechanism in the SBM equation.

**Figure 3:**
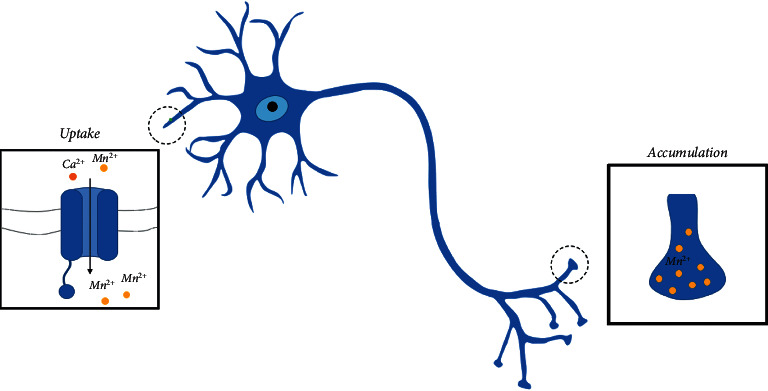
Mn ions neuronal pathway.

Therefore, Mn represents a valid allied to Gd in clinical imaging despite the fact that it also has some side effects. In fact, overexposure to free Mn ions could lead to a neurodegenerative disorder known as “manganism” [51]. A key role in the development of this disease is the similarity between Mn and Ca and Mg. Transport mechanisms for calcium can be used by Mn ions to enter the nervous system and then inside cells (see Figure 3). The result is the influence of normal synaptic transmission, in particular in the brain [50]. Mehdizadeh et al. investigated the behaviour *in vivo* of manganese oxide NPs, highlighting how they can interact with the nervous system leading to tau protein folding and neural death [67]. Despite this drawback related to the use of Mn, a renewed interest in a branch of MRI called MEMRI (Manganese-enhanced MRI) has grown as a technique for three specific uses: to obtain functional information about the brain or heart, to trace neuronal activity and axonal transport rates and to enhance brain anatomy and cytoarchitecture [68–70]. Indeed, unlike Gd which remains extracellular, the Mn influx and intracellular accumulation through voltage-gated calcium channels can be used to monitor changes in organ conditions giving back signal intensities that depend on cellular density. In particular, manganese chloride, Mn*Cl*_2_, is used more than other Mn-based CAs because maximal contrast is reached quickly after administration due to rapid uptake of free Mn and rapid removal from the blood.

In the case of accumulation into the brain, Mn ions can move along neuronal pathways allowing the enhancement in specific areas of the brain [68, 71–73]. In particular, AIM-MRI (activity-induced manganese-enhanced MRI) is a technique that involves pharmacological or somatosensory stimulations to have an increased specific brain activity after Mn-based CA injection [68, 74]. In this way, it is possible to detect impaired intracellular transport along axons typical of neurodegenerative diseases. AIM-MRI has been applied to the heart as well. About heart, a different Mn uptake by cardiac myocytes could be used to obtain a measure of calcium influx, which has a key role in myocardial contraction, highlighting cardiac inotropic impairments [75–77]. For example, Hu et al. investigated changes in signal intensity due to the presence of agents able to increase or reduce calcium influx, dobutamine, and diltiazem, respectively, which influenced Mn *Cl*_2_ uptake consequently [75]. Nevertheless, MEMRI has been applied only in preclinical studies due to the high doses needed and the unspecificity of current Mn-based CAs to translate it on humans [68].

## 5. Challenges and Perspectives

Magnetic resonance represents an optimal tool to gather anatomical and physiological information avoiding radiation-based diagnostic techniques. The need to use contrast agents is frequently required by clinicians in order to obtain clearer data from images and, consequently, about patient condition. In particular, the widespread and lasting use of Gadolinium-based CAs is explained by its excellent properties, which improve image quality. However, a risk of metal ions deposition in the brain, kidneys, and other organs has been highlighted in many studies, but, currently, additional strategies to improve safety and relaxivity of MRI CAs are under development. Indeed, different strategies based on, *Geometrical confinement, and Hydrodenticity* have been proposed and, in particular, the combination of Gd-CAs with hydrogel nanocarriers has proved to reduce toxicity and, at the same time, boost the relaxivity, providing also higher specificity due to the active and passive targeting abilities. Moreover, manganese-based contrast agents for MRI have been recently gaining interest and have been rationally designed to complex manganese in a manner that simultaneously provides high relaxivity and resistance to manganese dissociation. The exploitation of the role of manganese combined with nanotechnologies is promising and under investigation.

In the end, despite the advent of High Field MRI and Artificial Intelligence, the development of safer and more efficient contrast agents is still needed. Gadolinium and manganese, thanks to their excellent magnetic properties, still represent two valid opportunities to develop a new class of nanostructured contrast agents able to overcome drawbacks associated with actually used complexes. In particular, their combination with biopolymer matrices seems to be the right direction to obtain an equilibrium between image boost and reduced side effects.
